# Tackling skin lightening’s harms

**DOI:** 10.2471/BLT.23.020623

**Published:** 2023-06-01

## Abstract

A project in Gabon, Jamaica and Sri Lanka to eliminate mercury in skin-lightening products is highlighting the challenges faced in achieving that aim. Tatum Anderson reports.

Dr Heather Brown may be used to assessing the damage caused by skin-lightening soaps and creams, but that familiarity does not make the task any easier.

“It’s actually painful to see what people have done to themselves, sometimes,” she says, citing harms that range from rashes and discoloration to fungal infections that arise because the skin’s defences have been broken down.

A dermatologist at the St Jago Park Health Centre in Spanish Town, Jamaica, Brown is also the National Coordinator of Dermatology Services and Leprosy Control at Jamaica’s Ministry of Health and Wellness, and thus has a good overview of what is going on nationwide.

“The harms associated with these products are well documented,” she says, pointing to a 2018 nationwide lifestyle poll that revealed that just over 1 in 10 of the island nation’s 2.8 million inhabitants have used skin-whitening products, “and yet people continue to use them, many as part of a daily routine.”

Those harms derive from the products’ different constituents, notable among them inorganic mercury salts – chemical compounds comprising mercury and other substances such as chlorine.

“The damage done by the inorganic mercury salts used in skin-lightening products goes way beyond the harm done to skin,” explains Lesley Onyon, head of the World Health Organization’s (WHO) Chemical Safety and Health unit in the Department of Environment, Climate Change and Health.

“Once absorbed, they can cause significant damage to the kidneys and the nervous system. There is also evidence of an association with increased anxiety, depression and even psychosis.”

As Onyon is quick to point out, the damage is not limited to the individual using the product: “People need to understand that they are harming others,” she says. “For example when creams or skin-lightening soaps are washed off.”

Of particular concern is the risk of mercury released into the environment reacting with microbes to become methylmercury, the most toxic of the mercury compounds. “There is a significant danger of methylmercury bioaccumulation in fish which, when eaten, can impact children’s neurological development,” Onyon explains. “So, while skin lightening may be a personal choice, it has an impact on others.”

Certainly, it is a choice that many people make, as reflected by the robust industry that has grown up to meet demand. Reliable data are lacking, but analysts typically assign a worldwide value of around 10 billion US dollars to the sector currently, with an expectation of a 50% increase in value by the end of the decade.

“Skin lightening may be a personal choice [but] it has an impact on others.”

Lesley Onyon

The products are manufactured, bought and used worldwide, but their use is particularly widespread in countries in the African and South-East Asia Regions, as well as in the Region of the Americas.

Amira Adawe, founder and executive director of the Beautywell Project, a nongovernmental organization committed to ending skin lightening, recently returned from a research trip to Ethiopia, Djibouti, Kenya and Somalia, where she was surprised to find the products being sold even in resource-poor rural locations.

“I saw places where the only stores were those selling skin-lightening products,” she says. “I met women who were ready to go without basic essentials in order to pay for those products.”

Adawe discovered that most of the women felt that they had to use the products to improve their chances of getting a job and/or finding a partner. “Many of the women I interviewed had been told that men are only interested in lighter-skinned women, or see their employment and economic status affected by their dark colour,” she explains. “The predominance of white or lighter-skinned models, celebrities, and actors in films and on TV, in magazines and in advertising, only reinforces these beliefs.”

Addressing such pressures, and the discrimination that lies behind them (Adawe refers to it as ‘colourism’), is a core aim of a new, three-country, three-year initiative designed to eliminate mercury from skin-lightening products that was launched in February 2023.

With financial support from the Global Environment Facility (GEF) and technical support from WHO, the Biodiversity Research Institute (BRI), and the United Nations Environment Programme (UNEP) Global Mercury Partnership, Gabon, Jamaica and Sri Lanka have committed to reshaping policies, strengthening regulatory enforcement and raising awareness about the impact of the products on human and environmental health.

“The countries were chosen in recognition of the common challenges they face, and to ensure geographic diversity,” explains Ludovic Bernaudat, head of the Knowledge and Risk Unit at UNEP’s Chemicals and Health Branch, noting that skin-lightening products account for the bulk of the mercury in circulation in Gabon, Jamaica and Sri Lanka.

Jamaica and Sri Lanka are also both producers and significant consumers of skin- lightening products, offering opportunities to develop end-to-end mercury elimination strategies.

The hope is that by leveraging high-level political engagement and a determination to bring about change, the project will result in a roughly 50% reduction of mercury released into the environment in the three countries – 0.0043 tons per year in Gabon, 1.395 tons per year in Jamaica and 1.5456 tons per year in Sri Lanka.

Beyond this quantitative aspiration, the project is expected to generate valuable lessons regarding the challenges faced and ways to meet them that can be applied to countries worldwide.

As signatories to the Minamata Convention on Mercury – an international treaty aimed at reducing mercury pollution – each of the countries has already carried out a Minamata Initial Assessment (MIA), designed to determine the extent of mercury pollution and evaluate the effectiveness of existing policies and measures to address it.

“The MIAs have really helped the countries to get a clear idea of the challenges they face,” says Anil Sookdeo, the technical lead for chemicals and waste at the GEF.

Those challenges start with establishing credible baseline numbers for the presence of mercury in skin-lightening cosmetics. According to Dr Inoka Suraweera, a consultant community physician in the Environmental and Occupational Health Directorate at the Sri Lankan health ministry, Sri Lanka plans to collect and test 300 samples over a 2-year period with the support of staff from the Biodiversity Research Institute.

“It’s going to take us a while to undo some of these practices.”

Heather Brown

“Skin-lightening products that are above the one-part-per-million (1 ppm) limit agreed under the Minamata Convention will go through a second round of sampling to establish the range levels detectable in different brands,” Suraweera explains.

Suraweera would like to go faster, but the country lacks capacity for testing cosmetic products and for the biomonitoring needed to establish the levels of mercury in people’s blood, urine, hair and nails. Dr Ange Mibindzou Mouelet, chief executive officer of the Medicines Agency of Gabon, raises similar concerns, and says there are plans to invest in new capacity.

Without a solid baseline and knowledge of the mercury content of cosmetics on the market, monitoring the impact of interventions such as increased regulatory enforcement is going to be a challenge. So is effective public health messaging.

“We can’t just say, ‘There’s this harmful ingredient, you have to be careful,’” says Suraweera. “We need to know the numbers.”

On the regulatory front, the picture is mixed: each country has Minamata-based regulations in place but, to date, enforcement has been lacklustre. In Jamaica, Brown would like to see more inspection of shops and markets. “Products are often sold under the counter, some of them well in excess of the 1 ppm Minamata limit,” she says.

Challenges to regulating imports only compound the problem, something that affects not just the island states of Jamaica and Sri Lanka, but also Gabon, which has land borders with three countries and 885 kilometres of coastline. According to Bernaudat, there are plans to equip and train customs officers in the three countries with instruments that can detect mercury in higher-concentration products.

Onyon believes that in addition to targeting mercury levels in skin-lightening products, discouraging their use should be prioritized. “Just focusing on mercury may lead to it being replaced by other dangerous melanin-blockers,” she says. “What’s needed is to also reduce demand for the products in the first place.”

How that is to be done remains to be seen. For Sookdeo, addressing legacy norms is crucial. “The underlying socio-historical drivers of these practices need to be confronted and addressed if we are to make any progress on eliminating the use of these products and the mercury contained in them,” he says – a view shared by Bernaudat and Adawe, the latter putting particular emphasis on the need to address attitudes to skin colour.

“Simply telling people that creams are dangerous for them and their children, doesn’t necessarily change behaviour,” she says. “We started out with that kind of messaging and people were not listening. But then we shifted to more of a combined health education and dismantling colourism message, and we have seen better results.”

Heather Brown applauds such initiatives, and says that any efforts to reduce the demand for skin-lightening products will have to be sustained beyond the three-year duration of the project. “This is messaging that is going to need to continue for years to come,” she says. “It’s going to take us a while to undo some of these practices.”

**Figure:**
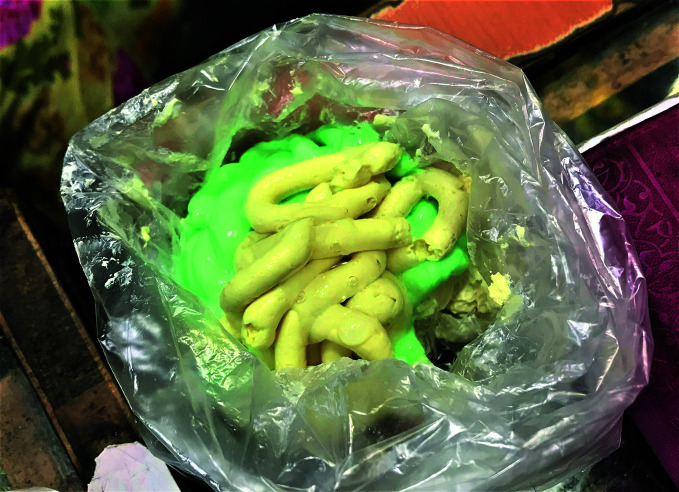
A skin-lightening mixture prepared in a store in Mogadishu, Somalia

Courtesy of Beautywell Project

**Figure:**
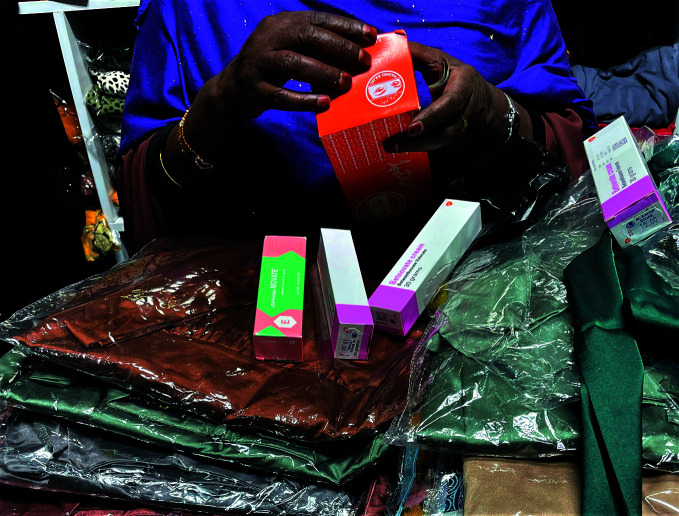
Skin-lightening products on sale in a store in Nairobi, Kenya

Courtesy of Beautywell Project

